# Brain glucose metabolism and gray matter volume in retired professional soccer players: a cross-sectional [ ^18^ F]FDG-PET/MRI study

**DOI:** 10.1055/s-0043-1768666

**Published:** 2023-05-31

**Authors:** Mateus Rozalem Aranha, Artur Martins Coutinho, Camila de Godoi Carneiro, Bruno Fraccini Pastorello, Adalberto Studart-Neto, Carla Cristina Guariglia, Miriam Harumi Tsunemi, Everton Luis Santos Moreira, Jéssica Natuline Ianof, Renato Anghinah, Ricardo Nitrini, Giovanni Guido Cerri, Juan Fortea, Carlos Alberto Buchpiguel, Claudia Costa Leite

**Affiliations:** 1 Universidade de São Paulo, Faculdade de Medicina, Instituto de Radiologia, São Paulo SP, Brazil.; 2 Universidade de São Paulo, Faculdade de Medicina, Centro de Medicina Nuclear, São Paulo SP, Brazil.; 3 Universidad Autónoma de Barcelona, Institut de Recerca, Hospital de la Santa Creu i Sant Pau, Facultad de Medicina, Barcelona, Spain.; 4 Universidade de São Paulo, Departamento de Neurologia, Faculdade de Medicina, São Paulo SP, Brazil.; 5 Universidade Estadual Paulista, Departamento de Bioestatística, Instituto de Biociências, Botucatu SP, Brazil.; 6 Fundación Catalana de Síndrome de Down, Barcelona, Spain.; 7 Centro de Investigación Biomédica en Red Enfermedades Neurodegenerativas, Madrid, Spain.

**Keywords:** Soccer, Brain Injuries, Traumatic, Chronic Traumatic Encephalopathy, Positron-Emission Tomography, Magnetic Resonance Imaging, Futebol, Lesões Encefálicas Traumáticas, Encefalopatia Traumática Crônica, Tomografia Por Emissão De Pósitrons, Imageamento Por Ressonância Magnética

## Abstract

**Background** Professional soccer athletes are exposed to repetitive head impacts and are at risk of developing chronic traumatic encephalopathy.

**Objective** To evaluate regional brain glucose metabolism (rBGM) and gray matter (GM) volume in retired soccer players (RSPs).

**Methods** Male RSPs and age and sex-matched controls prospectively enrolled between 2017 and 2019 underwent neurological and neuropsychological evaluations, brain MRI and [18F]FDG-PET in a 3.0-Tesla PET/MRI scanner. Visual analysis was performed by a blinded neuroradiologist and a blinded nuclear physician. Regional brain glucose metabolism and GM volume were assessed using SPM8 software. Groups were compared using appropriate statistical tests available at SPM8 and R.

**Results** Nineteen RSPs (median [IQR]: 62 [50–64.5] years old) and 20 controls (60 [48–73] years old) were included. Retired soccer players performed worse on mini-mental state examination, digit span, clock drawing, phonemic and semantic verbal fluency tests, and had reduced rBGM in the left temporal pole (pFDR = 0.008) and the anterior left middle temporal gyrus (pFDR = 0.043). Semantic verbal fluency correlated with rBGM in the right hippocampus, left temporal pole, and posterior left middle temporal gyrus (p ≤ 0.042). Gray matter volume reduction was observed in similar anatomic regions but was less extensive and did not survive correction for multiple comparisons (pFDR ≥ 0.085). Individual [18F]FDG-PET visual analysis revealed seven RSPs with overt hypometabolism in the medial and lateral temporal lobes, frontal lobes, and temporoparietal regions. Retired soccer players had a higher prevalence of *septum pellucidum* abnormalities on MRI.

**Conclusion** Retired soccer players had reduced rBGM and GM volume in the temporal lobes and *septum pellucidum* abnormalities, findings possibly related to repetitive head impacts.

## **Resumo**

**Antecedentes** Jogadores profissionais de futebol estão expostos a impactos cranianos repetitivos e ao risco de desenvolver encefalopatia traumática crônica.

**Objetivo** Avaliar o metabolismo glicolítico cerebral regional (MGCr) e o volume de substância cinzenta (vSC) em jogadores de futebol aposentados (JFAs).

**Métodos** Jogadores de futebol aposentados masculinos e controles pareados por idade e sexo foram incluídos prospectivamente entre 2017 e 2019. Foram realizadas avaliações neurológica e neuropsicológica, ressonância magnética (RM) e [18F]FDG-PET cerebrais (3.0-Tesla PET/RM). As imagens foram analisadas visualmente por um neurorradiologista e um médico nuclear cegos ao grupo de cada participante. O metabolismo glicolítico cerebral regional e o vSC foram avaliados através do programa SPM8. Os grupos foram comparados através de testes estatísticos apropriados disponíveis em SPM8 e R, de acordo com a distribuição e o tipo dos dados.

**Resultados** Dezenove JFAs (mediana [IIQ]: 62 [50–64.5] anos) e 20 controles (60 [48–73] anos) foram incluídos. Os JFAs tiveram pior desempenho no mini-exame do estado mental e nos testes de dígitos, desenho do relógio, fluência verbal e fluência semântica e apresentaram MGCr significativamente reduzido no polo temporal e no giro temporal médio anterior esquerdos. Fluência semântica (animais) apresentou correlação positiva com MGCr no hipocampo direito, no polo temporal esquerdo e no aspecto posterior do giro temporal médio esquerdo. Menor vSC foi observado nas mesmas regiões, porém este achado não sobreviveu à correção para comparações múltiplas. Análise individual do [18F]FDG-PET cerebral revelou sete JFAs com claro hipometabolismo nas faces medial e lateral dos lobos temporais, nos lobos frontais e nas regiões temporoparietais. Os JFAs apresentaram ainda maior prevalência de anormalidades do septo pelúcido.

**Conclusão** Os JFAs apresentam MGCr e vSC reduzidos nos lobos temporais, além de anormalidades do septo pelúcido, achados possivelmente relacionados a impactos cranianos repetitivos.

## INTRODUCTION

Chronic traumatic encephalopathy (CTE) is a neurodegenerative disease related to repetitive head trauma 1 first described in boxers 2 and mostly studied in soldiers, 3 4 American football players, 5 6 7 and fighters. 8 The neuropathological hallmark of CTE is the deposition of phosphorylated Tau protein (pTau) in neurons, and astroglia with perivascular distribution in the depth of sulci. 9 Deposition of TAR DNA-binding protein 43 kDa (TDP-43) and, less frequently, amyloid-β can also be found. 9

Neuroimaging studies of the long-term effects of traumatic brain injury have shown that individuals exposed to repetitive head impacts have reduced cortical glucose metabolism and gray matter volume with the regional distribution depending on the mechanism, intensity, and frequency of the head impacts in the exposed subjects. 10 11

Previous studies have shown that soccer players had worse cognitive performance on neuropsychological tests than controls 12 and have mortality related to neurodegenerative diseases 3.45 times higher than in the general population. 13 Moreover, recent post-mortem studies demonstrated CTE's neuropathology in demented retired soccer players (RSPs). 14 15 16 These data suggest that soccer athletes, who are exposed to long-term, repetitive head impacts are at risk of developing CTE.

Soccer is among the most practiced sports worldwide, and yet soccer players remain an understudied population regarding the long-term effects of sport-related repeated head impacts on the brains of these athletes. The main purpose of the present cross-sectional observational study is to investigate multimodal neuroimaging findings ([18F]FDG-PET and magnetic resonance imaging [MRI]) in RSPs.

## METHODS

### Selection of participants

Male RSPs and healthy age- and sex-matched controls were prospectively enrolled between January/2017 and September/2019. The present study was approved by the Research Ethics Committee of the São Paulo University Medical School (registry 1.561.037). All participants provided informed consent.

The RSPs were randomly sampled among the athletes registered in a local sports association (the syndicate of athletes of the state of São Paulo) or randomly referred by the orthopedics department of our university hospital, irrespective of any cognitive complaints. The inclusion criteria for the RSP group were: male sex, and previous professional soccer practice. Inclusion criteria for controls were: male sex and lack of traumatic brain injury (TBI) (control group). Common exclusion criteria for RSPs and controls were: previous neurological disease (unrelated to neurodegeneration), contraindications to MRI, 17 incidental intracranial lesions on MRI, or limiting imaging artifacts. Besides these common exclusion criteria, it was considered not eligible for the study any RSP who presented a history of TBI resulting in hospitalization and any control with regular amateur/recreational soccer practice.

Controls were recruited from the families of the RSPs and as volunteers at our university hospital's neurology department.

### Neurological evaluation

Participants underwent neurological examination by a board-certified neurologist. The standard neurological assessment included a physical examination and a clinical interview which addressed the presence of any cognitive complaints, history of TBI, TBI-related loss of consciousness, neurologic, cardiovascular, and endocrine diseases, and a neuropsychological evaluation with the Mini-Mental State Examination (MMSE), 18 digit span (forward and backward), 19 figure memory test (naming, recognition, incidental memory, immediate memory, learning, delayed recall), 20 verbal fluency (phonemic and semantic), 21 22 and clock drawing 23 tests. 24 Participants were screened for traumatic encephalopathy syndrome (TES) based on Montenigro's criteria. 25 Lumbar puncture for the analysis of CSF biomarkers was proposed to all participants.

### Image acquisition

[18F]FDG-PET and MRI images were simultaneously acquired in a 3.0-Tesla PET/MRI scanner (Signa, GE Healthcare, Boston, MA, USA). The MRI protocol included volumetric T1-weighted (T1WI), T2-weighted (T2WI), fluid-attenuated inversion recovery (FLAIR), and susceptibility-weighted angiography (SWAN) images. Metabolic images were acquired 30 minutes after intravenous [18F]FDG injection. The imaging acquisition protocol is detailed in the Supplementary Material (online only).

### Image processing

The GM volume was assessed with voxel-based analysis using Statistical Parametric Mapping (SPM) 8 software (Wellcome Department of Human Neuroimaging). Initially, skull and extracranial structures were manually extracted from T1WI using MRIcron software (McCausland Center for Brain Imaging). Then, skull-striped T1WI were spatially normalized into an anatomic template and segmented into CSF, GM, and WM using the *Diffeomorphic Anatomical Registration using Exponentiated Lie algebra* (DARTEL) algorithm. Images were then modulated by the Jacobian determinant and adjusted to the Montreal Neurological Institute (MNI) coordinates. Besides quantifying GM volume, this pipeline created the study-specific anatomic template for [18F]FDG-PET processing.

For [18F]FDG-PET group analysis, images were co-registered with their respective T1WI (to correct for partial volume effects [PVEs], as described by Meltzer et al. 26 ) and spatially normalized using SPM8 into the study-specific anatomic template previously generated with DARTEL. Scans were smoothed with an 8.0-mm full width at half maximum Gaussian filter to improve signal-to-noise ratio and mitigate misregistration into the template space. A default threshold of 0.8 of the mean uptake inside the brain was selected to ensure that the analysis included only voxels mapping cerebral tissue. Global uptake differences between scans were adjusted using a proportional scaling approach (global mean) at SPM8.

WMH in the FLAIR images were segmented automatically with the Lesion Growth Algorithm 27 as implemented in the Lesion Segmentation Tool toolbox (version 1.2.3 2013–03–12, www.statisticalmodelling.de/lst.html ) for SPM, using a threshold of 0.3, as recommended by the developer. 27

### Visual analysis

[18F]FDG-PET images were evaluated by a neuroradiologist and a nuclear physician, both experienced in neurologic [18F]FDG-PET and blinded to the participant's group. Scans were rated as “normal,” “abnormal,” or “borderline” (nonspecific findings, possibly within normal limits), based on visual interpretation assisted by the 3D-SSP semiquantitative software (CortexID Suite, GE Healthcare, Boston, MA, USA) as previously proposed. 28 In addition to subjective visual analysis, readers considered a Z-score < 2.0 in at least 2 cortical areas, after normalization for the pons and the cerebellum, as a reference of abnormality.

To investigate typical patterns of Alzheimer disease (AD) 28 or other neurodegenerative diseases, 29 the regions of reduced rBGM in all abnormal scans were detailed.

T1WI was inspected by a neuroradiologist to detect structural neuroimaging abnormalities related to repetitive head impacts, namely *cavum septum pellucidum* (CSP), cavum vergae (CV) and *septum pellucidum* fenestration (SPF). Also, brain atrophy was assessed on T1WI with the Global Cortical Atrophy, Medial Temporal Lobe Atrophy, Posterior Atrophy, Anterior Cingulate Atrophy, Orbitofrontal Atrophy, Anterior-Temporal Atrophy, and Fronto-Insular Atrophy scales. 30 31 The scales were rated separately for each hemisphere and averaged before statistical analysis.

FLAIR and SWAN images were assessed for detection of WMH according to the Fazekas scale, 32 and for detection of microbleeds and superficial siderosis, respectively.

### Statistical analysis

Demographic, clinical data, and MRI findings on visual analysis were statistically analyzed using R ( https://www.r-project.org/ ). For group comparison, categorical data were assessed with the chi-squared test and numerical data with the *t* -test for independent sample or the Mann-Whitney U-test, according to data distribution assessed with the Shapiro-Wilk test. Data with a normal distribution are expressed as mean ± standard deviation (SD), and data with a nonnormal distribution are expressed as median; interquartile range (IQR). The threshold for significance was set at *p* = 0.05.

For the initial exploratory analyses of [18F]FDG-PET and T1WI, statistical parametric maps of [18F]FDG uptake and GM volume were generated using SPM8 with the threshold for significance at the voxel level set at *p*_uncorrected_ = 0.001 (Z-score = 3.09) with a minimum extension of 10 voxels in the corresponding cluster. Results were considered valid when surviving correction for multiple comparisons with the false discovery rate (FDR) method (pFDR ≤ 0.05). 33

Relevant peak voxels from the statistical parametric maps were initially identified in the Montreal Neurological Institute (MNI) coordinate system and then converted to the Talairach and Tournoux coordinates with the MNI2Tal web application (Legacy BioImage Suite). 34

Numeric values (measured in kBq/ml) representing the mean [18F]FDG uptake for each participant (a proxy for regional brain glucose metabolism [rBGM] in the clusters with statistically significant results in the SPM group analysis) were obtained with the MarsBar toolbox for SPM ( http://marsbar.sourceforge.net/). ^35^ These values were used to investigate correlations of [18F]FDG uptake with the time of soccer practice and the scores of neuropsychological tests in the RSPs using linear regressions.

## RESULTS

### Participant characteristics

Nineteen male RSPs and 20 healthy and age-matched male controls were included ( Supplementary Figure S1 [online only]).

The median age was 62 (50–64.5) and 60 (48–73) years old, and the duration of formal education was 14 (11–15) and 15 (11.8–16) years in the RSP and control groups, respectively. No significant age differences were found between groups; however, controls had higher educational levels ( Table 1 ). The RSPs had a total soccer practice time of 19.7 ± 6.2 years. Regarding playing position, 10/19 (52.7%) RSPs played in defense, 4/19 (21.0%) in midfield, and 5/19 (26.3%) in offensive roles.

**Table 1:** Demographics, MRI visual analysis, clinical and neuropsychological data, and burden of white matter FLAIR hyperintensities in both groups

			Retired soccer players (19, male)	Controls (20, male)	*p* -value
Demographics (years, median; IQR)	Age (years old)		62; 50–64.5	60; 48–73	0.527 ^a^
	Education		14; 11–15	15; 11.8–16	0.035 ^a^
Septum pellucidum abnormalities (% of participants)	Cavum Septum Pellucidum		68	15	0.001 ^b^
	Cavum Vergae		37	20	0.243 ^b^
	Fenestration of Cavum Septum Pellucidum		32	0	0.006 ^b^
Brain atrophy (visual rating) (median; IQR)	Global cortical atrophy (Pasquier) scale		1; 0–1	1; 0–2	0.136 ^a^
	Medial temporal lobe atrophy (Scheltens) scale		0; 0–1	0;0–0.125	0.446 ^a^
	Posterior atrophy (Koedam) scale		0; 0–1	0; 0–1	0.543 ^a^
	Anterior cingulate atrophy scale		0; 0–0.5	0; 0–1	0.414 ^a^
	Orbitofrontal atrophy scale		0; 0–0	0; 0–0	0.964 ^a^
	Anterior-temporal atrophy scale		0; 0–0	0; 0–1	0.471 ^a^
	Frontoinsular atrophy scale		0; 0–0	0; 0–0.25	0.730 ^a^
White matter FLAIR hyperintensities (visual rating and quantification) (median; IQR)	Fazekas scale		1; 0–1	1; 0–1	0.666 ^a^
	Volume of white matter FLAIR hyperintensities ^d^		5.7; 2.4–32.5	2.7; 1.5–18.1	0.224 ^a^
Neuropsychological evaluation	MMSE total score (median; IQR)		27; 25.5–29	29; 28.8–30	0.003 ^a^
	Clock drawing test (median; IQR)		9; 9–10	10; 9–10	0.036 ^a^
	Semantic verbal fluency test (animals) (mean ± SD)		15.7 ± 4.4	20.8 ± 4.8	0.002 ^c^
	Phonemic verbal fluency test total score* (mean ± SD)		35.3 ± 11.5	42.6 ± 9.6	0.040 ^c^
	Digit span test total score* (mean ± SD)		8.3 ± 2.1	11.5 ± 3.9	0.005 ^c^
	Figure memory test	Naming (median; IQR)	10; 10–10	10; 10–10	1.000 ^a^
		Incidental memory (mean ± SD)	6.5 ± 2.4	6.0 ± 2.1	0.467 ^c^
		Immediate memory (median; IQR)	8; 7–9	8; 7–9	1.000 ^a^
		Learning (median; IQR)	9; 8–10	9; 8–10	0.609 ^a^
		Delayed recall (median; IQR)	9; 7–9	9; 8–9.3	0.437 ^a^
		Recognition (median; IQR)	10; 9.5–10	10; 10–10	0.094 ^a^
Clinical evaluation	History of traumatic brain injury		11 (58%)	0	<0.001 ^c^
	Psychiatric symptoms		6 (31%)	2 (10%)	0.171 ^b^
	Subjective cognitive complaints		2 (10%)	5 (25%)	0.447 ^b^
	Arterial systemic hypertension		4 (21%)	6 (30%)	0.562 ^b^
	Type 2 diabetes		2 (10%)	2 (10%)	0.920 ^b^

Abbreviations: IQR, interquartile range; MMSE, Mini-Mental State Examination; SD, standard deviation.

Notes: ^a^ Mann-Whitney U test. ^b^ Chi-squared test. ^c^ t-test for independent samples. ^d^ Volume of WM FLAIR hyperintensities measured with Lesion Segmentation Tool toolbox for SPM 8 software (threshold = 0.30). Data with normal distribution are expressed as mean ± SD and data with non-normal distribution are expressed as median; IQR. *One control (with normal MMSE, naming, incidental memory, immediate memory, learning, delayed recall, and recognition scores) had missing values on the Digits Span and Phonemic Verbal Fluency Tests.

### *History of head trauma*

All RSPs were exposed to frequent heading. However, 11/19 (58%) reported TBI related to head-to-head (9/11, 82%), head-to-ground (1/11, 9%), and head-to-elbow (1/11, 9%) impacts. Loss of consciousness was reported by 3/19 (16%) players. Traumatic brain injury was reported by 4/10 (40%) defenders, 2/4 (50%) midfielders, and ⅗ (60%) offenders ( *p* = 0.689).

### *Neurological evaluation*

Compared with controls, RSPs had significantly lower MMSE scores, performed significantly worse on semantic verbal fluency, clock drawing, phonemic verbal fluency, and digit span tests ( Table 1 ). The figure memory test showed no significant differences between groups.

Among RSPs, 6/19 (31%) reported anxiety, depression, attention deficits, or alcohol abuse, while 2/20 (10%) of controls reported anxiety or depressive symptoms. Cognitive decline, defined as an impairment in sporadic memory, spatial orientation, or verbal fluency (after ruling out non-neurodegenerative causes), was clinically confirmed in 2/19 (10%) RSPs, diagnosed with TES and further classified as probable CTE according to Montenigro's criteria. 25 Among controls, 4/20 (20%) had subjective cognitive complaints that were not confirmed as cognitive decline in the neurological evaluation ( Table 1 ).

### *[18F]FDG-PET group analysis*

**Figure 1:**
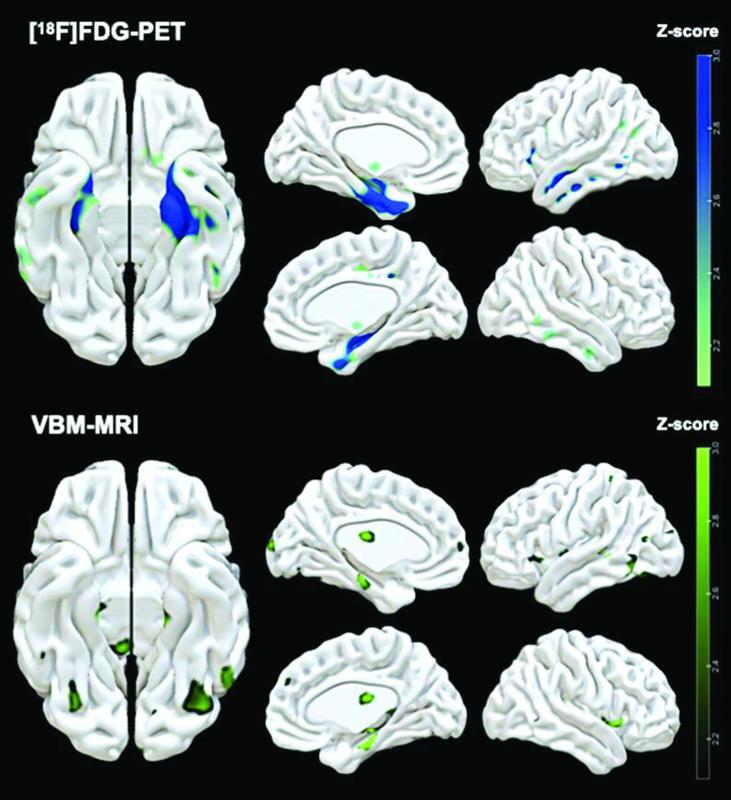
Notes: Maps generated by Surf Ice software ( http://www.nitrc.org/projects/surfice/ .) with *p* <0.05, uncorrected. Bars on the right: Zscores ranging from *p* = 0.05 (Z-score = 2.0) to *p* = 0.001 (Z-score = 3.0). Reduced [ ^18^ F]FDG uptake is observed in the left temporal pole, the anterior left middle temporal gyrus, the right hippocampus, the posterior left fusiform/inferior temporal gyrus, the left insula, and the right parahippocampal gyrus. Reduced GM volume is observed in the right parahippocampal gyrus, the posterior left middle temporal gyrus, and the posterior left fusiform/inferior temporal gyrus. Illustrative anatomic localization of the peak clusters of reduced rGBM and reduced GMvolume in retired soccer players compared with controls.

The RSPs exhibited reduced [18F]FDG uptake in the left temporal pole (pFDR = 0.008) and in the anterior left middle temporal gyrus (pFDR = 0.043). Smaller clusters of glucose hypometabolism were observed in the right hippocampus, the posterior left fusiform/inferior temporal gyrus, the left insula, and the right parahippocampal gyrus but did not survive correction for multiple comparisons ( Figure 1 , Supplementary Table S1 [online only]). These findings were observed after correction for PVE and persisted when age and education were covariates. The FDG uptake in the right hippocampus was negatively correlated with the soccer practice time ( *p* = 0.039; r _Pearson_ = - 0.48). No correlation between soccer practice time and [18F]FDG uptake was observed in the remaining clusters of hypometabolism.

**Figure 2:**
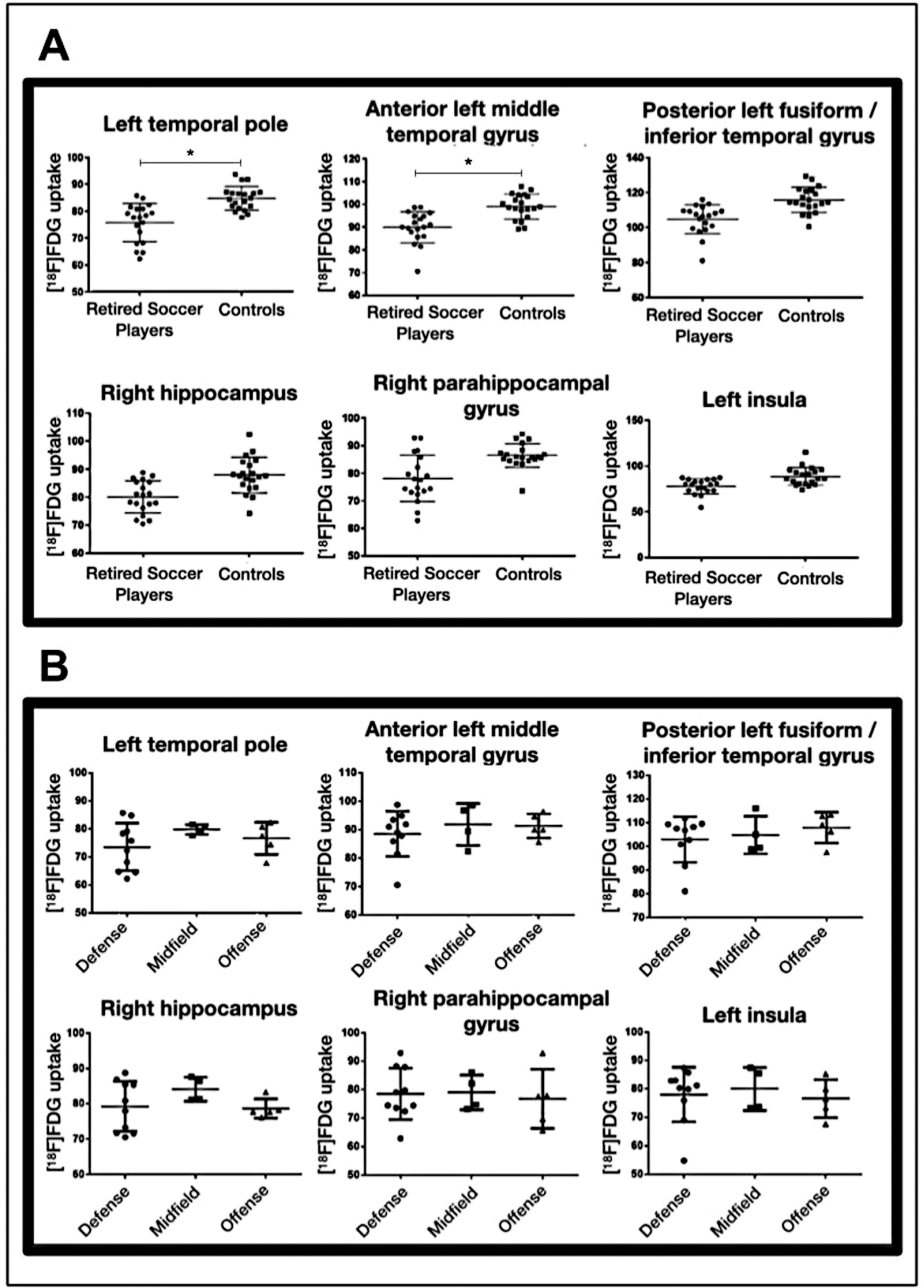
Note: Plots generated with Prism 6 software ( www.graphpad.com/scientific-software/prism/ ). Scatter plot of individual [ ^18^ F]FDG uptake in the clusters of reduced rBGM for each participant ( **A** ) and for each retired soccer player, compared regarding their playing position ( **B** ). ( **A** ). Individual [ ^18^ F]FDG uptake in all clusters is consistently lower in retired soccer players than in controls, with statistically significant differences between groups in the left temporal pole and the anterior leftmiddle temporal gyrus(*). The three individuals with the lowest uptake in the left temporal pole were defensive players (participants **A** , **B** , and **C** ). Participants A and B were clinically classified as possible and probable CTE, respectively. ( **B** ). No significant differences were observed among defenders, midfielders, and offenders regarding [ ^18^ F]FDG uptake in these areas ( *p* < 0.216).

The individual rBGM in all clusters of reduced [18F]FDG uptake was consistently lower in RSPs than in controls, mostly evident in the left temporal pole, the anterior left middle temporal gyrus, the posterior left fusiform/inferior temporal gyrus, and the right hippocampus ( Figure 2A ). However, no significant differences in rBGM were observed among playing positions ( Figure 2B ).

Scores of the Semantic Verbal Fluency (animals) test correlated positively with [18F]FDG uptake in the right hippocampus ( *p* = 0.006; r ^2^_Pearson_ = 0.61), left temporal pole ( *p* = 0.042; r ^2^_Pearson_ = 0.47), and in the posterior left middle temporal gyrus ( *p* = 0.041; r ^2^_Pearson_ = 0.47). No significant correlation was observed between the remaining neuropsychological test scores and the [18F]FDG uptake.

### [18F]FDG-PET visual analysis

**Figure 3:**
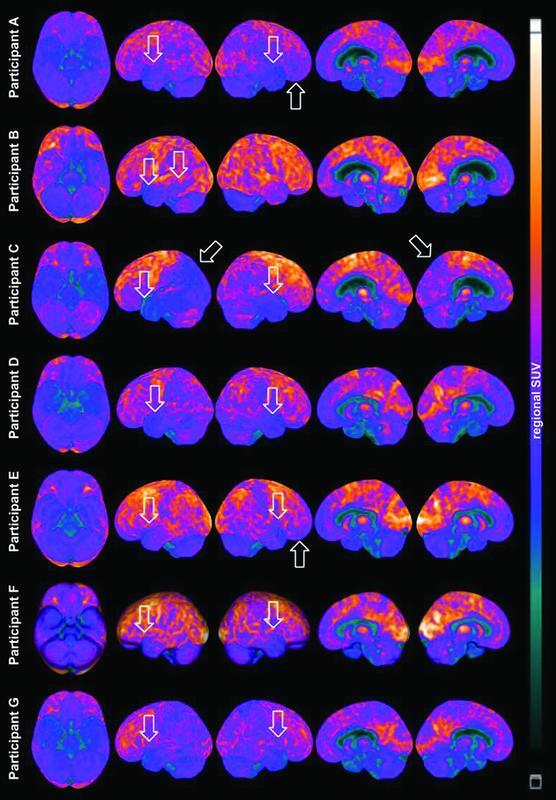
Abbreviation: SUV, Standard Uptake Value. Individual 3D-SSP of the [ ^18^ F]FDG-PET metabolic images from all seven retired soccer players with definitely abnormal scans in the visual analysis. All seven retired soccer players classified as abnormal presented with reduced [18F]FDG-PET uptake in the lateral temporal lobes, with some individual variation. Participants A and E have also reduced rBGM in the frontal lobes, and participant C in the left temporal-parietal-occipital region and the left precuneus.

Abnormal [18F]FDG-PET scans were observed in 16/19 (84%) RSPs and in only 4/20 (20%) controls ( *p* < 0.001). Clearly abnormal exams were found in 7/19 (36%) RSPs (participants A-G, Figure 3 ), while 9/19 (47%) had borderline scans. Only 1/20 (5%) controls had an abnormal exam, and 3/20 (15%) had borderline alterations. None of the RSP who reported a loss of consciousness presented with abnormal scans.

All RSPs with abnormal scans showed hypometabolism in the medial and lateral temporal lobes. Additionally, participants A and E had reduced rBGM in the frontal lobes, and participant C in the left temporal-parietal-occipital region, extending to the ipsilateral precuneus. Participants C, D, and G had temporoparietal hypometabolism that could be visually interpreted as AD. However, given the extension to occipital regions or the lack of clear involvement of the posterior cingulate gyrus and precuneus, these findings did not fit the typical AD pattern (Figure 3 and Supplementary Table S2 [online only]). The only control with abnormal [18F]FDG-PET had reduced rBGM in both cerebellar hemispheres.

The RSPs with abnormal [18F]FDG-PET were not significantly different from those with borderline and normal scans (analyzed together) regarding age (63.1 ± 3.8 versus 54.4 ± 12.7 years old, *p* = 0.098), education (11.6 ± 3.8 versus 13.3 ± 2.3 years, *p* = 0.245), and time of soccer practice (19.3 ± 7.6 versus 20 ± 5.6 years, *p* = 0.817).

The RSPs with abnormal [18F]FDG-PET on visual analysis presented with lower MMSE scores than RSPs with normal or borderline scans (p _Mann-Whitney_ = 0.045). No differences between RSPs with abnormal and normal or borderline [18F]FDG-PET scans were observed regarding the remaining neuropsychological tests.

### MRI visual analysis

Cavum septum pellucidus and SPF were significantly more frequent in RSPs (68%) than in controls (15%). No differences between groups were observed regarding the frequency of CV ( Table 1 ).

Regarding scores of brain atrophy and Fazekas scales, no differences between RSPs and controls were observed ( Table 1 ). Visual analysis of SWAN images was unremarkable in all participants.

### GM volume analysis

The RSPs exhibited reduced GM volume in similar brain regions as those with reduced [18F]FDG uptake, including the right parahippocampal gyrus (pFDR = 0.544), the posterior left middle temporal gyrus (pFDR = 0.085), and the posterior left fusiform/inferior temporal gyrus (pFDR = 0.085) (Figure 1, Supplementary Table S1 [online only]). None of these clusters survived correction for multiple comparisons.

### Quantitative assessment of WMH

The quantitative analysis revealed no significant differences in the volume of WMH between RSPs (5.7; 2.4–32.5) and controls (2.7; 1.5–18.1) ( *p* = 0.224).

## DISCUSSION

The present cross-sectional observational study investigated multimodal neuroimaging findings in retired professional soccer players (RSPs). We found that RSPs exhibited reduced glucose metabolism in the temporal lobes, with clusters in the left temporal pole and the anterior left middle temporal gyrus surviving correction for multiple comparisons. They also presented smaller clusters of reduced GM volume in similar anatomic regions that did not survive correction for multiple comparisons. The areas of hypometabolism were also detected in a visual analysis by experts. Ultimately, these findings point to neurodegeneration in the temporal lobes, with a slight predominance on the left side, and could be related to long-term repetitive head impacts.

These results agree with the previous neuroimaging and neuropathological features observed by Grinberg et al. in an RSP with a clinical diagnosis of late-onset AD. 14 In a post-mortem 3T MRI, these authors found atrophy in the anterior and medial structures of the temporal lobes (greater on the left) and CSP. Also, the neuropathological examination showed phospho-tau CTE pathology in the temporal lobes and limbic structures, as well as TDP-43-related hippocampal sclerosis. 14 Therefore, signs of neurodegeneration in the temporal lobes were expected in our study.

Several studies with military personnel, boxers, and American football players, 4 8 10 36 37 38 39 have shown mild TBI-related hypometabolism in the cerebellum, the pons, the temporal and frontal regions, the posterior cingulate, and the thalamus. In RSPs, however, the clusters of cortical hypometabolism are less extensive and widespread than reported in those populations. These imaging findings likely reflect differences in the type, intensity, and frequency of head impacts to which soccer players are exposed.

Lesman-Segev et al. 10 showed that, when compared with controls, American football players with TES and negative amyloid-PET have clusters of reduced FDG uptake in the medial temporal lobe structures and frontal cortex (with minor involvement of the lateral left temporal and parietal lobes) and clusters of reduced GM volume in frontal regions, the insula, and anterior temporal lobes. We did not observe hypometabolism or reduced GM volume in frontal areas; however, the involvement of lateral and medial temporal lobe structures in RSPs without involving areas typically affected in AD (precuneus and posterior cingulate gyrus) is in agreement with those findings in American football athletes.

A study by Meabon et al. 39 demonstrated a dose-response relationship between blast-related concussion and cerebellar hypometabolism in veteran soldiers (subjects exposed to more blast-related head impacts had lower cerebellar glucose uptake). Despite differences in the spatial distribution of glucose hypometabolism clusters (likely related to different trauma mechanisms, intensity, and frequency in different populations), we observed that lower rBGM in the right hippocampus is related to longer careers among RSPs, pointing to a possible dose-response relationship between sports-related mild TBI and brain hypometabolism in these athletes.

On the MRI visual analysis, we found a higher prevalence of CSP and SPF in RSPs than in controls. Similarly, Koerte et al. 40 and Lesman-Segev et al. 10 found a higher frequency of CSP in American football athletes. These findings have been reported as a CTE feature 40 41 and are likely related to the thinning and detachment of the septum pellucidum layers caused by the impact of cerebrospinal fluid (CSF) waves generated during the head trauma. 41

Regarding WMH, a study by Berginström et al., 42 using an automated segmentation method to quantify these lesions, demonstrated that the burden of WMH increases with the TBI severity but that no differences are observed between mild TBI patients and healthy controls. In our work, we used the same approach as Berginström et al. 42 to segment WMH, and we found no differences in the load of WMH between RSPs and controls, which was expected since RSP were exposed to mild, but not moderate or severe, TBI.

The main limitation of our study is the lack of Tau-PET imaging (regionally unavailable) and CSF biomarkers since all participants refused lumbar puncture. In the absence of CSF biomarkers, tau, and amyloid-PET, we could not exclude other causes of neurodegeneration/neurodegenerative diseases, namely AD.

However, the regional pattern of hypometabolism observed in RSPs was not suggestive of AD pathology. Besides, the neuropsychological evaluation showed that RSPs had lower global cognitive performance, with impaired attention and executive functions. No impairment of episodic memory was observed in this group. This neuropsychological profile would be expected in CTE, 25 but not in AD. 43 44 Also, given the mean age of RSP (62 years old, IQR: 50–64.5), if the hypometabolism in RSPs were related to AD pathology, it would be of the pre-senile AD, which is rarer than the sporadic form. We believe it unlikely that our random recruitment resulted in a cohort of RSPs with a predominance of pre-senile AD or even frontotemporal lobar degeneration.

Although, to our knowledge, this is the largest sample of RSPs with multimodal brain PET/MRI to date, the relatively small number of RSPs limited the sub-analysis regarding the different playing positions and risk of CTE.

The present paper shows that [18F]FDG-PET/MRI can be used to investigate athletes with suspected CTE, including using a visual clinically-based approach at the individual level. Additionally, we demonstrated that RSPs have brain metabolic and structural changes in the temporal lobes and a higher prevalence of CSP and SPF, findings similar to those reported in other athletes and possibly related to long-term repetitive head impacts. Further studies with larger samples, CSF biomarkers, tau, and amyloid-PET will deepen our understanding of this condition.

In conclusion, RSPs have reduced regional brain glucose metabolism in the temporal lobes and a higher prevalence of CSP and SPF than age and sex-matched controls. Also, the cerebral glucose hypometabolism in RSP may present a dose-response relationship with the career length of the RSP. These findings might be related to chronic brain damage due to repetitive head impacts related to sportive practice.
