# Supplementary material for: Brain glucose metabolism and gray matter volume in retired professional soccer players: a cross-sectional [ 18 F]FDG-PET/MRI study
Source: Arq Neuropsiquiatr. 2023 May 31;81(5):433–43. doi: 10.1055/s-0043-1768666 (PMC10232036; doi:10.1055/s-0043-1768666)
Supplement: Supplementary file 1 — Supplementary Material [file 10-1055-s-0043-1768666-s220186.pdf]

Supplementary Material

Image Acquisition

[18F]FDG-PET and MRI images were simultaneously acquired in a 3.0-Tesla PET/MRI scanner (Signa, GE Healthcare).

MRI protocol included T1-weighted images (T1WI, SPGR acquisition, voxels of $1.0 \times 1.0 \times 1.0$ mm, flip angle [FA] = 8, repetition time [TR]/echo time [TE] = 8/3 milliseconds, 196 sagittal slices), T2-weighted images (T2WI, CUBE technique, voxels of $1.0 \times 1.0 \times 1.0$ mm, FA = 90, TR/TE = 2500/88 milliseconds, 196 sagittal slices), fluid attenuated inversion recovery (FLAIR) images (CUBE technique with inversion pulse with TI = 1905 milliseconds, voxels of $1.3 \times 1.3 \times 1.3$ mm, FA = 90, TR/TE = 6500/141 milliseconds, 152 sagittal slices), and susceptibility-weighted angiography (SWAN) images (gradient-echo strongly weighted in $T2^*$, voxels of $0.94 \times 0.94 \times 2.0$ mm, TE = 38 milliseconds, TR = 52 milliseconds, FA = 10, 78 axial slices).

Metabolic images were acquired with a matrix of 256×256 and an acquisition time of 15 minute, starting 30 minute after intravenous injection of 5.0 mCi (185 MBq) of [18F]FDG, produced in the on-site cyclotron of our university hospital (PETtrace 880, GE Healthcare). PET images were then reconstructed on a dedicated workstation, using an interactive protocol (OSEM, 28 subgroups, and 04 interactions for each scan) and a standardized processing algorithm (VUE Point FX), and smoothed using a cut-off value of 3.0 mm. Data were corrected for scattering, attenuation, and radioactive decay.

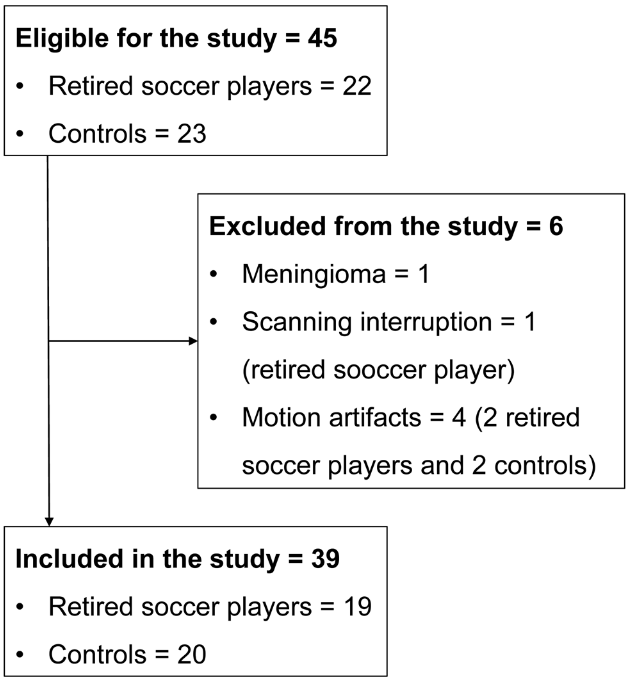

Supplementary Fig. S1 Flowchart shows the number of participants included in the study.

 © 2023. Academia Brasileira de Neurologia. All rights reserved.

Supplementary Table S1 SPM8 statistics: clusters of reduced [^{18}F]FDG uptake and GM volume

Set-level		Cluster-level				Peak-level					MNI coordinates			BA
p	c	p_{FWE}	p_{FDR}	k_E	p_{uncorr}	p_{FWE}	p_{FDR}	k_E	$Z_{(E)}$	p_{uncorr}	mm	mm	mm	
Reduced [^{18}F]FDG uptake														
**0.353**	**8**	**0.006**	**0.008**	**497**	**0.001**	**0.360**	**0.370**	**4.55**	**4.03**	**0.000**	**-22**	**8**	**-38**	**38**
						0.410	0.370	4.48	3.98	0.000	-22	-2	-36	36
						0.684	0.370	4.15	3.73	0.000	-32	-8	-36	20
		**0.069**	**0.043**	**262**	**0.011**	**0.471**	**0.370**	**4.40**	**3.92**	**0.000**	**-52**	**-16**	**-20**	**21**
						0.669	0.370	4.17	3.75	0.000	-46	8	-26	38
						0.787	0.370	4.02	3.64	0.000	-50	0	-20	38
		**0.644**	**0.310**	**69**	**0.155**	**0.636**	**0.370**	**4.21**	**3.78**	**0.000**	**-54**	**-56**	**-4**	**37**
						0.855	0.380	3.92	3.56	0.000	-54	-46	-18	37
		**0.227**	**0.103**	**159**	**0.039**	**0.680**	**0.370**	**4.15**	**3.74**	**0.000**	**32**	**-18**	**-20**	**54**
						0.873	0.380	3.89	3.54	0.000	26	-14	-24	54
		**0.930**	**0.530**	**24**	**0.398**	**0.751**	**0.370**	**4.07**	**3.67**	**0.000**	**26**	**4**	**-30**	**36**
		**0.813**	**0.402**	**44**	**0.251**	**0.980**	**0.645**	**3.59**	**3.31**	**0.000**	**-26**	**24**	**4**	**13**
		**0.986**	**0.733**	**8**	**0.642**	**0.992**	**0.768**	**3.48**	**3.22**	**0.001**	**-56**	**-30**	**-20**	**20**
		**0.996**	**0.839**	**8**	**0.839**	**0.998**	**0.948**	**3.35**	**3.11**	**0.001**	**10**	**-38**	**38**	**23**
Reduced GM volume														
**0.901**	**3**	**0.434**	**0.544**	**110**	**0.060**	**0.063**	**0.062**	**5.51**	**4.65**	**0.000**	**22**	**-22**	**-14**	**36**
		**0.957**	**0.085**	**27**	**0.333**	**0.055**	**0.845**	**4.10**	**3.68**	**0.000**	**-60**	**-38**	**0**	**21**
		**0.995**	**0.085**	**10**	**0.500**	**0.930**	**0.845**	**3.96**	**3.57**	**0.000**	**-42**	**-70**	**-14**	**19**

Abbreviations: [^{18}F]FDG, [^{18}F]fluorodeoxyglucose; BA, Brodmann area; FDR, False Discovery Rate; FEW, Familywise Error; GM, gray matter; k_E, cluster size (in number of voxels); MNI, Montreal Neurological Institute; p_{FDR}, *p*-value corrected for multiple comparisons using FDR method; p_{FWE}, *p*-value corrected for multiple comparisons using FWE method; p_{uncorr}, *p*-value uncorrected for multiple comparisons; $Z_{(E)}$, Z-score.

Supplementary Table S2 Characteristics of the seven retired soccer players who presented abnormal [^{18}F]FDG-PET scans on visual analysis (subjects A-G)

Subject	Age	Playing position	MMSE	Neurological evaluation	Hypometabolic areas on [^{18}F]FDG-PET visual analysis	TBI
A	63	Defensive	23	Possible CTE	Bilateral temporal lobes<br>Right frontal lobe	no
B	63	Defensive	22	Probable CTE	Left temporal lobe	yes
C	69	Defensive	21	Normal	Bilateral temporal lobes (more extensive on the left)<br>Left parieto-occipital region<br>Left precuneus	no
D	62	Defensive	28	Normal	Bilateral temporal lobes	no
E	58	Defensive	27	Normal	Bilateral temporal lobes (more extensive on the right)<br>Right frontal lobe	yes
F	60	Offensive	26	Normal	Bilateral temporal lobes, more extensive on the right)	yes
G	67	Offensive	28	Normal	Bilateral temporal lobes	no

Abbreviations: CTE, chronic traumatic encephalopathy; MMSE, Mini-Mental State Examination; TBI, traumatic brain injury.

 © 2023. Academia Brasileira de Neurologia. All rights reserved.
